# Grey mould of strawberry, a devastating disease caused by the ubiquitous necrotrophic fungal pathogen *Botrytis cinerea*

**DOI:** 10.1111/mpp.12794

**Published:** 2019-04-04

**Authors:** Stefan Petrasch, Steven J. Knapp, Jan A. L. van Kan, Barbara Blanco‐Ulate

**Affiliations:** ^1^ Department of Plant Sciences University of California, Davis Davis CA USA; ^2^ Laboratory of Phytopathology Wageningen University Wageningen Netherlands

**Keywords:** disease management, fruit ripening, fruit‐pathogen interaction, plant breeding, plant defence, primary infection, secondary infection

## Abstract

The fungal pathogen *Botrytis* *cinerea* causes grey mould, a commercially damaging disease of strawberry. This pathogen affects fruit in the field, storage, transport and market. The presence of grey mould is the most common reason for fruit rejection by growers, shippers and consumers, leading to significant economic losses. Here, we review the biology and epidemiology of the pathogen, mechanisms of infection and the genetics of host plant resistance. The development of grey mould is affected by environmental and genetic factors; however, little is known about how *B. cinerea* and strawberry interact at the molecular level. Despite intensive efforts, breeding strawberry for resistance to grey mould has not been successful, and the mechanisms underlying tolerance to *B. cinerea* are poorly understood and under‐investigated. Current control strategies against grey mould include pre‐ and postharvest fungicides, yet they are generally ineffective and expensive. In this review, we examine available research on horticultural management, chemical and biological control of the pathogen in the field and postharvest storage, and discuss their relevance for integrative disease management. Additionally, we identify and propose approaches for increasing resistance to *B. cinerea* in strawberry by tapping into natural genetic variation and manipulating host factors via genetic engineering and genome editing.

## Introduction

Strawberry (*Fragaria* × *ananassa*) is an important soft fruit crop that is grown worldwide on more than 370 000 hectares (FAO STAT, 2014) and, for the United States alone, the total value of the annual strawberry production exceeds US$2.3 billion (USDA, 2016). Strawberries are beneficial to the human diet as a source of macro‐ and micronutrients, vitamins and health promoting antioxidants (Basu *et al.*, 2014; Giampieri *et al.*, 2015; Wang and Lin, 2000).

Strawberry is a perennial herbaceous plant with short stems (crowns) and densely spaced leaves. Strawberry produces complex accessory and aggregate fruit composed of achenes and a receptacle (Darrow, 1966). Achenes are small single‐seeded fruit, whereas the receptacle is considered to be anatomically equivalent to floral meristem tissue (Hollender *et al.*, 2012). *F*. × *ananassa* is an allo‐octoploid (2n = 8x = 56) that originated as a synthetic hybrid between the octoploid species *Fragaria chiloensis *and *Fragaria virginiana* (Bringhurst, 1990; Edger *et al.* 2019; Darrow, 1966; Rousseau‐Gueutin *et al.*, 2008).

**Figure 1:**
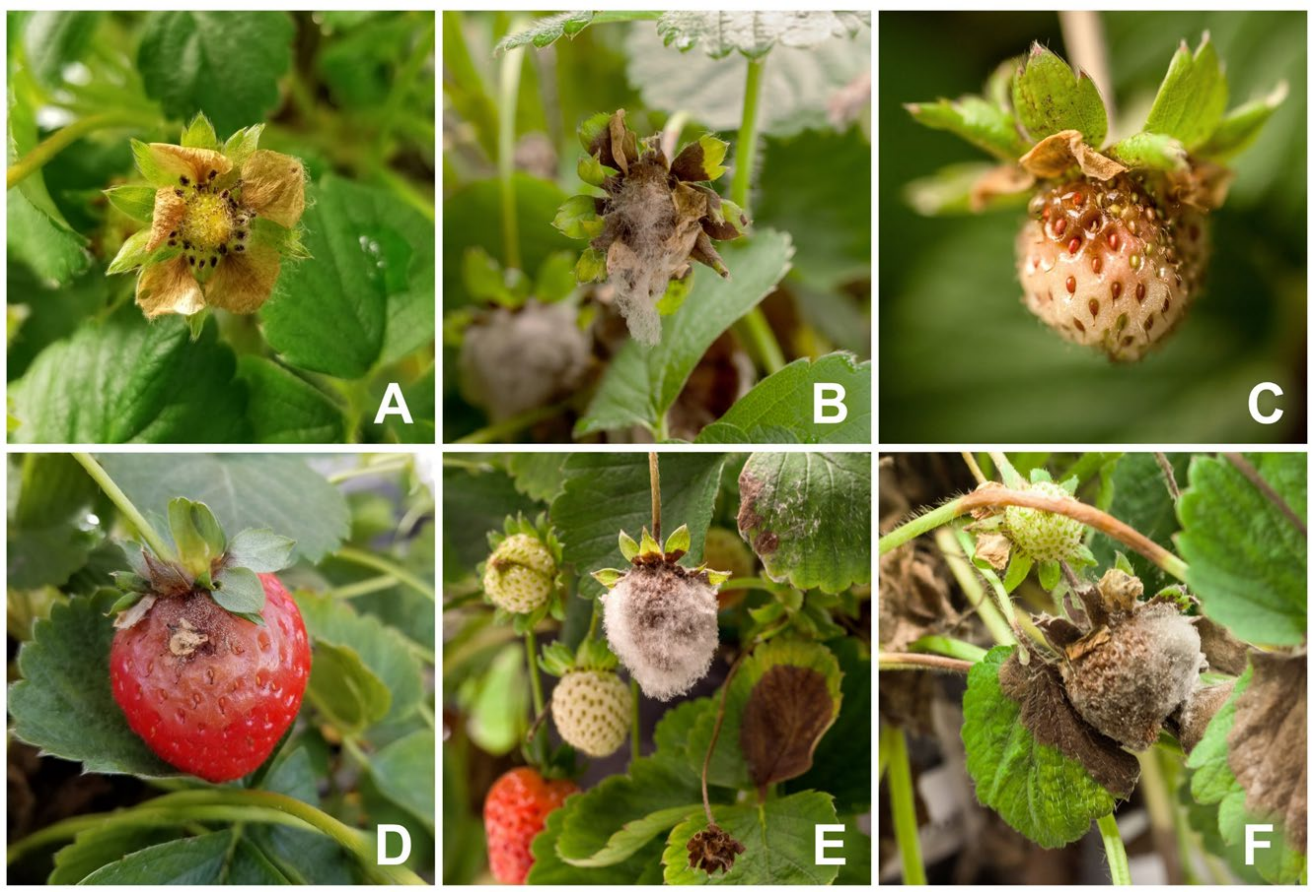
Symptoms of *Botrytis cinerea* infections in strawberry. Panel A shows a senesced flower with *B. cinerea* mycelium growth. Panel B shows an advanced floral infection. Panels C and D show infections of fruit at different stages. An infected petal can be seen as the source of fruit infection in Panel D. Browning of leaves due to *B. cinerea* infections is shown in Panels E and F.

Strawberry is affected by several pathogens including fungi, bacteria, viruses and nematodes. The most economically impactful pathogens of strawberry are fungi, which can infect all parts of the plant and cause severe damage or death (Garrido *et al.*, 2011). Amongst the fungal pathogens, the ascomycete *Botrytis cinerea* is considered the primary pathogen of harvested strawberries in the world leading to impactful economical losses to the strawberry industry. *B. cinerea* causes grey mould in fruit and senescing organs but can also affect vegetative tissues (Fig. 1). Under wet conditions, more than 80% of strawberry flowers and fruits can be lost if plants are not sprayed with fungicides (Ries, 1995).

## The Pathogen *Botrytis cinerea*

*B. cinerea* has no apparent host specificity and can infect more than 1000 plant species (Elad *et al.*, 2016). The pathogen is found worldwide and causes disease in many fruit, flower and leafy vegetable crops (Boff, 2001; Carisse, 2016; Elad *et al.*, 2007). *B. cinerea* is classified as a necrotroph, meaning that it prefers to infect and grow on damaged or senescing tissues, eventually causing tissue death. The inoculum (e.g. conidia) of the fungus is highly abundant and ubiquitous and usually comes from infected plant tissues (Jarvis, 1962). *B. cinerea* mainly enters the host via wounds or natural openings (Holz *et al.*, 2007). Infections of non‐senescing or unripe plant organs usually lead to limited damage and quiescent infections (Dewey and Grant‐Downton, 2016; Jarvis, 1962). Different types of quiescence have been described: (i) delay of conidia germination or growth arrest after germination (Jarvis, 1994), (ii) endophytic symptomless growth in the apoplast (Barnes and Shaw, 2003; Sowley *et al.*, 2010), (iii) colonization of abscising flower organs (e.g. petals) followed by growth into ovaries or receptacles where growth arrests (Bristow *et al.*, 1986). Independent of the type of infection, the pathogen generally enters a short asymptomatic, biotrophic phase at the beginning of the disease cycle (Veloso and van Kan, 2018). An aggressive necrotrophic phase commonly succeeds the quiescent or asymptomatic phase once plant organs start to senesce or ripen, during which *B. cinerea* causes rapid decay of the infected tissues (Elad *et al.*, 2007).

*B. cinerea*'s infection mechanisms have been studied in model organisms and further characterized thanks to the availability of high‐quality reference genome sequences (Amselem *et al.*, 2011; Van Kan *et al.*, 2017; Staats and van Kan, 2012). The fungus is known to actively promote plant susceptibility by employing a variety of virulence factors (Choquer *et al.*, 2007; Nakajima and Akutsu, 2014; Petrasch *et al.*, 2019). In early stages, *B. cinerea* deploys sRNAs and effector proteins to suppress premature host cell death and immune responses, which enables the fungus to establish inside the host and accumulate biomass prior to the necrotrophic phase (Veloso and van Kan, 2018). It was demonstrated that *B. cinerea* Dicer‐like proteins DCL1 and DCL2 produce sRNAs that are secreted from fungal hyphae and translocated to the plant cell where they interfere with the host RNAi mechanisms to silence host immune response genes in *Arabidopsis* and tomato leaves (Wang *et al.*, 2017b; Weiberg *et al.*, 2013).

Some secreted virulence factors can lead to host cell death, like effector proteins, toxins and enzymes involved in reactive oxygen species (ROS) production (Schumacher, 2016). *B. cinerea *can also secrete oxalic acid that lowers the pH of the host tissues and stimulates the production and activity of fungal enzymes like pectinases, laccases and proteases (Fernández‐Acero *et al.*, 2010; Manteau *et al.*, 2003; Prusky and Lichter, 2007; Sharon *et al.*, 2007). Furthermore, oxalic acid accumulation leads to Ca^2+^ chelation, which in turn weakens the pectin structures of plant cell walls and inhibits the deposition of callose (Chakraborty *et al.*, 2013). Other virulence factors are cell wall degrading enzymes (CWDEs) that enable *B. cinerea *to cause plant cell lysis and loosen walls to facilitate tissue penetration (Blanco‐Ulate *et al.*, 2016a). The fungus is known to produce plant hormones or hormone analogues that may disturb the host's cellular metabolism and immune responses. The relevance of these mechanisms for the capacity of *B. cinerea *to infect strawberry remains unknown.

## Strawberry‐*Botrytis cinerea* Pathosystem

**Figure 2:**
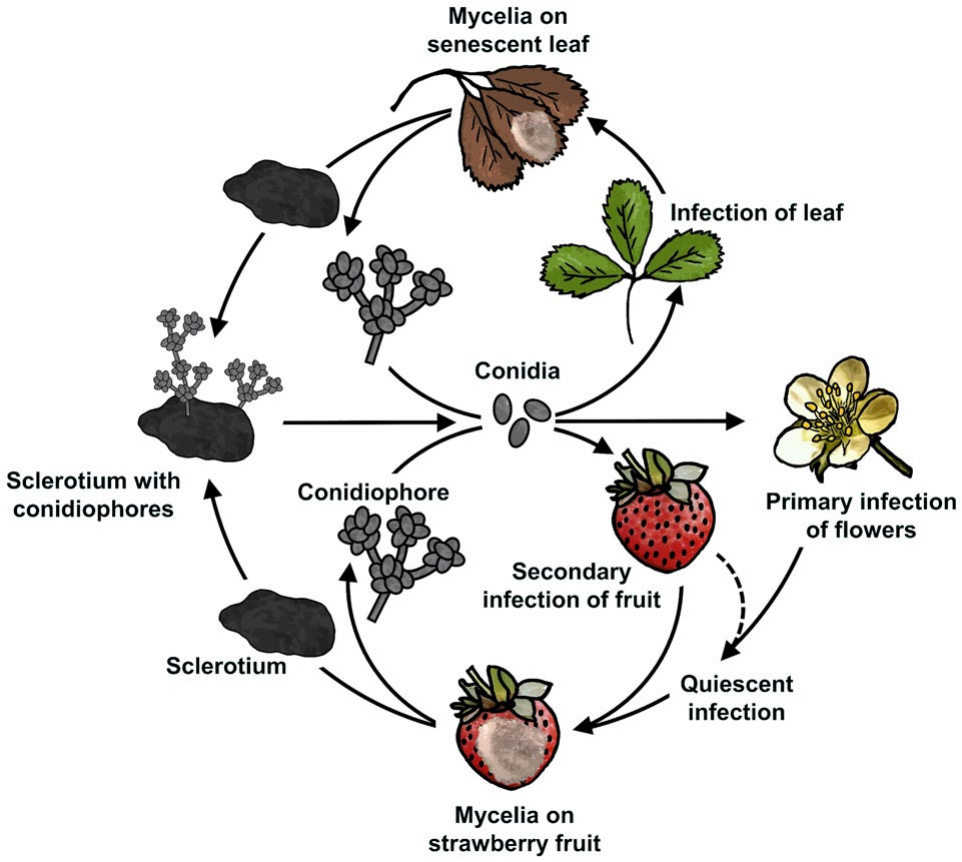
*Botrytis cinerea* disease cycle in strawberry. Sources of *B. cinerea* inoculum include infected leaves and sclerotia. Primary infections of flowers and secondary infections of fruit are depicted.

Grey mould in strawberries can result from *B. cinerea* infections of open flowers (primary infections) or by penetration of fruit receptacle tissues (secondary infections) (Bristow *et al.*, 1986). In primary infections, *B. cinerea *infects flower organs during or right after flowering, allowing hyphae to grow into the receptacle (Fig. 2). The sources of primary inoculum range from overwintering sclerotia to conidia or mycelium from infected neighbouring plants (Jarvis, 1962). Infected senescent petals, stamens and calyxes can facilitate primary infections in fruit (Powelson, 1960). Histological studies have shown that even though styles are frequently infected, fungal growth appears to be strongly inhibited and never reaches the receptacle. In contrast, fungal growth in colonized stamens can reach the receptacle in some cultivars (Bristow *et al.*, 1986).

Following infection of the unripe receptacle by *B. cinerea*, fungal growth is usually arrested and a symptomless quiescent phase occurs. The mechanisms that lead to quiescent infections are not yet fully understood. Proanthocyanins (PAs) appear to induce *B. cinerea* quiescence in unripe fruit by restricting the activity of fungal enzymes like polygalacturonases (PGs) that are necessary for aggressive infection of hosts (50% inhibition in unripe fruit compared to 8% inhibition in ripe fruit). Even though PA content in fruit remains constant during ripening, increasing polymerization of PAs leads to lower inhibitory activity in ripe fruit (Jersch *et al.*, 1989). Similarly, anthocyanins might delay *B. cinerea *infections or cause quiescence (van Baarlen *et al.* 2007). For instance, strawberries illuminated with white fluorescent light showed increased anthocyanin content and delayed development of grey mould (Saks *et al.*, 1996). Reduced fruit decay has also been observed in raspberries with high pigmentation (Harshman *et al.*, 2014) and in transgenic tomatoes that accumulate anthocyanins (Bassolino *et al.*, 2013; Zhang *et al.*, 2013). Other small phenolics, especially catechins, may have a role in quiescence. High levels of catechins inhibit fungal growth, and a decrease in catechins is correlated with a reduction of other antifungal compounds such as lipoxygenases (Prusky and Lichter, 2007). Interestingly, young and ripe fruit have low catechin concentration, suggesting that initial infections of young receptacles are possible because they do not yet accumulate enough catechins to stop colonization (Puhl and Treutter, 2008). *B. cinerea* quiescence is complex and involves additional factors besides the accumulation of phenolic compounds. It has been proposed that quiescence in unripe fruit is initiated by: (i) lack of nutrients such as sugars (e.g. mono‐ and disaccharides) from the host, (ii) presence of preformed antifungal compounds, (iii) unsuitable environment for fungal virulence factors (Prusky and Lichter, 2007). In unripe strawberries, factors from all three categories are present, including lack of available sugars (Knee *et al.*, 1977), preformed antifungal compounds (Hébert *et al.* 2002; Terry *et al.*, 2004), and high activity of PG‐inhibiting proteins (PGIPs) (Mehli *et al.*, 2005). Induction of the necrotrophic phase in ripe strawberries could be triggered by changes in biochemical composition of the host tissues associated with the ripening process, such as increased sugar content, volatile production and alteration of plant defences (Neri *et al.*, 2015; Prusky and Lichter, 2007). These modifications promote not only fungal growth but also host susceptibility, e.g. via the release of oxalic acid and efflux of toxins (Prusky and Lichter, 2007).

During secondary infections, the fungus initiates the necrotrophic phase without quiescence (Holz *et al.*, 2007). The sources of conidia for secondary infections can also be diverse, from senescent leaves to infected fruit (Fig. 2). Conidia from *B. cinerea*‐infected flower parts are major sources of secondary inoculum (Bristow *et al.*, 1986). It has been estimated that more than 64% of the strawberry infections result from organic fragments that are in contact with the fruit, such as petals and stamens (Fig. 1D; Jarvis, 1962). Contrary to other fruit (e.g. raspberries), senescent flower parts often adhere to strawberries long enough to retain water films for at least 8 h, which is the time needed for *B. cinerea* conidia germination (Jarvis, 1962).

**Figure 3:**
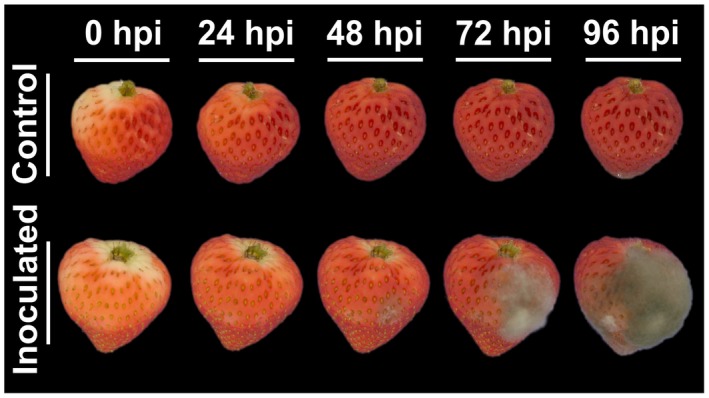
Progression of *Botrytis cinerea* infection in ripe strawberries. Inoculation was performed by wounding the fruit and adding a *B. cinerea *conidia suspension on the surface of the wound. Fruit are shown immediately after inoculation, and at 24 h to 96 h post‐inoculation (hpi). Wounded controls are included.

Secondary infections can also result from nesting, which corresponds to direct penetration of mycelia growing on neighbouring plant organs such as infected leaves and fruit (Fig. 1F; Braun and Sutton, 1988). Generally, secondary infections proceed rapidly and *B. cinerea *can complete its germination and infection as fast as 16 h post‐inoculation (hpi) with a rapid increase in fungal biomass at 48 hpi (Fig. 3; Mehli *et al.*, 2005). Early responses of strawberries to infection include higher expression of the defence genes *FaPGIP* and *FaChi 2‐1* (Class II Chitinase), whereas lower expression of the reference gene DNA Binding Protein – *FaDBP* indicates extensive cell death induced by *B. cinerea *at late stages of infection (Mehli *et al.*, 2005)*.*

## Relevance of Ripening Processes to *Botrytis cinerea* Infections of Strawberries

**Figure 4:**
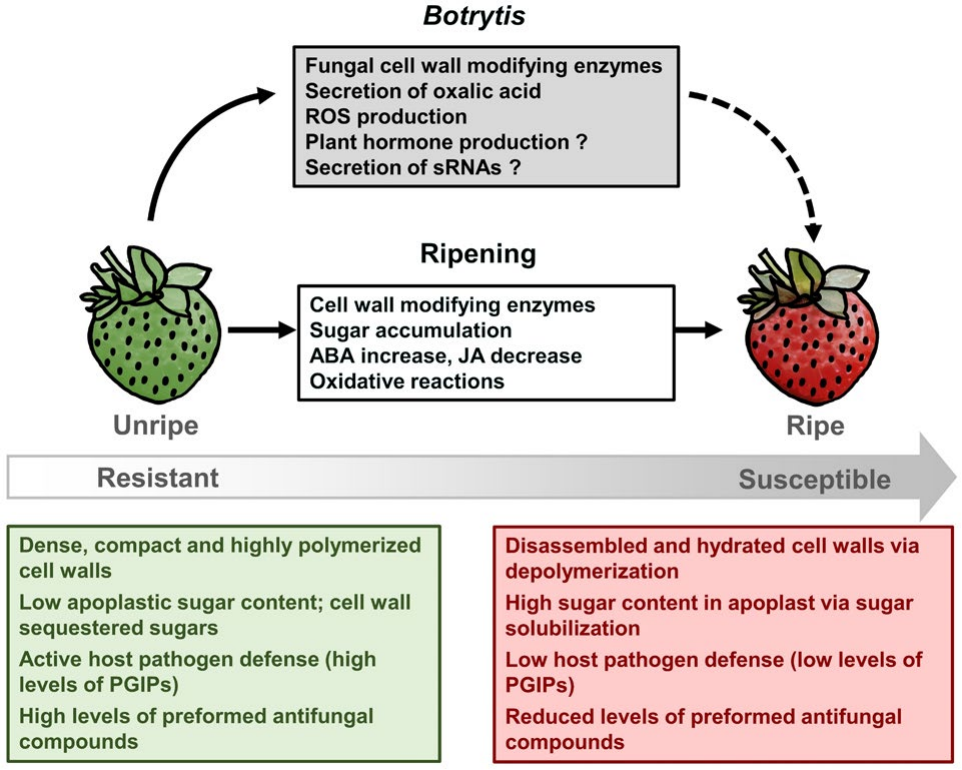
Ripening processes influence *Botrytis cinerea* infections of strawberries. Unripe fruit present unsuitable conditions for *B. cinerea* infection, while ripe fruit provide a favourable environment for pathogen growth. Pathogenicity factors are activated by *B. cinerea *during strawberry ripening and lead to increased susceptibility. ABA, abscisic acid; JA, jasmonic acid; PGIPs, PG‐inhibiting proteins; ROS, reactive oxygen species.

Fruit ripening influences the susceptibility of strawberry fruit to *B. cinerea* (Fig. 4). Strawberries are mostly resistant to infection in their unripe stage, where they restrict fungal growth by causing quiescence. However, in the ripe stage, strawberries are highly susceptible and decay rapidly. Fruit susceptibility to fungal disease increases as ripening progresses; hence, *B. cinerea *appears to promote susceptibility in unripe fruit by activating specific ripening‐related processes (Blanco‐Ulate *et al.*, 2016b). In tomato fruit, master transcriptional regulators of ripening have been shown to have different roles in disease susceptibility. For example, the activity of the tomato transcription factor NON‐RIPENING (NOR) favours *B. cinerea* infection (Cantu *et al.*, 2009). Strawberries are non‐climacteric fruit with a ripening programme different from that of climacteric tomatoes. Thus, a deeper understanding of strawberry ripening regulation and how *B. cinerea *may modulate particular ripening events are pivotal to characterize the dynamics of the strawberry‐*B. cinerea* pathosystem.

Recent transcriptomic studies of developing strawberries point out that ripening events start between the ‘large green’ and ‘white’ stages, and involve changes in cell wall composition, sugar metabolism, hormone biosynthesis and responses, pigmentation and antioxidant levels (Guo *et al.*, 2018; Sanchez‐Sevilla *et al.*, 2017; Wang *et al.*, 2017a). Moreover, a general decrease of oxidative phosphorylation processes has been observed during strawberry ripening (Sanchez‐Sevilla *et al.*, 2017; Wang *et al.*, 2017a). Normal strawberry ripening involves a variety of biochemical and physiological processes, some of which are discussed below in the context of *B. cinerea* interactions.

### Cell wall modifications

Ripening is associated with the disassembly of the fruit cell walls, which leads to tissue softening. Cell wall degradation benefits *B. cinerea* as it reduces mechanical barriers to infection and spread, increases the possibilities of bruising (e.g. leading to more wounds for pathogen entry) and provides the fungus with access to simple sugars as a carbon source (Blanco‐Ulate *et al.*, 2016b; Blanco‐Ulate *et al.*, 2016a; Brummel and Harpster, 2001).

In strawberry, cell wall solubilization occurs early in fruit development when the walls start to swell (Knee *et al.*, 1977). Cell wall solubilization correlates with an increase in fruit sugar content, resulting from polysaccharide breakdown. A decrease of acid‐soluble pectins and the alcohol‐insoluble fraction of cell walls occur during ripening, whereas the water‐soluble content increases (i.e. enriched in non‐covalent bound pectins). The degree of pectin solubilization and depolymerization is highly‐related to strawberry fruit firmness (Rosli *et al.*, 2004). Silencing of an endogenous pectin lyase (PL) gene in strawberry resulted in fruit with higher external and internal firmness, mostly due to low pectin solubilization, stiffer cell walls, and increased cell to cell adhesion (Jimenez‐Bermudez *et al.*, 2002; Santiago‐Domenech *et al.*, 2008). Besides PL, other enzymes that may have affected strawberry firmness include PGs, β‐galactosidases, endoglucanases, α‐arabinofuranosidases and β‐xylosidases (Figueroa *et al.*, 2010).

In addition to the fruit endogenous cell wall disassembly, *B. cinerea* secretes an extensive array of CWDEs that target most polysaccharides in the fruit cell walls, particularly pectins (Blanco‐Ulate *et al.*, 2016a). These CWDEs include fungal PGs, such as *Bcpg2*, a gene that is mainly active in the early penetration stage (Mehli *et al.*, 2005). The expression of *B. cinerea* PGs is dependent on the host species, the plant tissue, temperature and the stage of infection (Blanco‐Ulate *et al*. 2014; ten Have *et al.*, 2001).

### Cuticle changes

Another barrier for *B. cinerea* infection is the fruit cuticle. During fruit expansion and ripening the cuticle gets thinner, which makes strawberries more susceptible to initial penetration by germinating conidia. *B. cinerea* can penetrate the plant cuticle by secretion of cutinases (van Kan, 2006). Additionally, cuticle properties can result in higher incidence of cracks and other damages through which *B. cinerea* can enter the fruit without the need of cutinases (Holz *et al.*, 2007). Studies on strawberry cuticles are scarce and only exist for leaf tissues (Kim *et al.*, 2009). In tomato fruit, thicker and stiffer cuticles lead to higher resistance to initial *B. cinerea* infections. Moreover, it is known that the chemical composition of the cuticle changes during tomato ripening, and this is likely to be the case in strawberry (Isaacson *et al.*, 2009; Kosma *et al.*, 2010).

### Sugar accumulation

During ripening, the content of sugar in strawberries increases and therefore can serve as nutrients for *B. cinerea*. In unripe strawberries, the main sugars are glucose and fructose with low concentrations of sucrose. Sucrose levels increase rapidly during de‐greening and red colouring (Jia *et al.*, 2011). In tomato, it has been shown that the *Cnr* mutant, which does not accumulate high levels of sugars is still highly susceptible to *B. cinerea *infection (Blanco‐Ulate *et al.*, 2016b). This observation suggests that even though sugars may serve as a susceptibility factor, high sugar concentrations are not essential for *B. cinerea *infection. However, sugar content could still influence susceptibility to *B. cinerea* as specific sugars may serve as ripening initiation signals. For instance, sucrose regulates abscisic acid (ABA) levels in strawberries, which are necessary for normal ripening and could influence fruit susceptibility as described below (Blanco‐Ulate *et al.*, 2016b; Jia *et al.*, 2011; Li *et al.*, 2011). Like other ripening‐related events, *B. cinerea *can alter neutral sugar and sugar acid levels in the infected host tissues, mainly by degradation and depolymerization of cell walls. This was reported for infections in tobacco and *Arabidopsis* leaves, where the fungus degrades pectins to release the monosaccharide galacturonic acid (Zhang and van Kan, 2013).

### Plant hormone biosynthesis and signalling

ABA is the main hormone regulating and inducing ripening in strawberries (Jia *et al.*, 2016; Li *et al.*, 2011). ABA biosynthesis during fruit ripening is triggered by a decrease in pH, turgor changes, sugar accumulation, and the switch of sugars from mainly glucose and fructose to sucrose (Jia *et al.*, 2011; Li *et al.*, 2011). Effects of ABA on strawberry susceptibility to fungal disease have not been extensively studied, but down‐regulation of the ABA biosynthetic gene β‐glucosidase *FaBG3* has been reported to result in fruit with limited ripening and higher *B. cinerea *resistance (Li *et al.* 2013). In tomato, ABA accumulation is related to higher pathogen susceptibility, probably via activation of senescence (Blanco‐Ulate *et al.*, 2013; Harrison *et al.*, 2011; Lee *et al.*, 2011). During strawberry ripening, the increase of ABA is correlated with a decrease of auxin, which induces early fruit growth and expansion but is known to inhibit ripening processes (Jia *et al.*, 2011). The role of auxin in fruit susceptibility seems to depend on the plant species, as indole acetic acid (IAA) treatment in *Arabidopsis* leads to susceptibility, whereas IAA‐treated tomato leaves and eggplant fruit show lower infection severity (Savatin *et al.*, 2011; Sharon *et al.*, 2007). Ethylene has a secondary organ‐specific role in strawberry ripening, particularly in achenes and green and white receptacles (Knee *et al.*, 1977; Merchante *et al.*, 2013). Ethylene increases the susceptibility of tomato to *B. cinerea* by inducing ripening; however, its functions during strawberry infections are yet to be fully characterized. ABA, IAA and ethylene accumulation are altered by polyamine levels, which are positively correlated with fruit susceptibility to *B. cinerea* during strawberry ripening (Guo *et al.*, 2018). Other hormones, such as brassinosteroids (BRs) and jasmonic acid (JA) are present at lower levels during strawberry ripening. BR positively regulates vitamin C levels, sugar and anthocyanin biosynthesis during ripening, while negatively regulating acidity and concentration of other phenolic compounds (Ayub *et al.*, 2018). JA acts synergistically with ethylene by activating its biosynthesis in strawberries (Mukkun and Singh, 2009). Endogenous JA levels are modulated by methyl jasmonate (MeJA) and the JA carboxyl methyltransferase that lead to high levels in white fruit and a decline during ripening, antagonistically to ABA (Garrido‐Bigotes *et al.*, 2018; Preuß *et al.*, 2014). In strawberry, JA appears to be involved in defence responses against *B. cinerea*. For example, strawberries treated with MeJA had a delayed and much slower progression of *B. cinerea* infections (Saavedra *et al.*, 2017; Zhang *et al.*, 2006).

## Hijacking of Ripening Regulation by *Botrytis cinerea*

As indicated previously, *B. cinerea* releases enzymes and metabolites that act as virulence factors but may also induce plant responses that are beneficial for fungal infection (van Kan, 2006). A relevant example of the manipulation of physiological processes in the host by *B. cinerea *is the interference with specific developmental processes. In tomato plants, *B. cinerea* infections modified host gene expression to increase susceptibility, such as the induction of senescence in leaves (Swartzberg *et al.*, 2008). Moreover, infected unripe tomato fruit show premature expression of genes involved in ethylene synthesis during tomato ripening (Blanco‐Ulate *et al.*, 2013; Cantu *et al.*, 2009). These findings suggest that *B. cinerea* could initiate ethylene production and thereby stimulate early ripening. As strawberries are non‐climacteric fruit, ethylene production of *B. cinerea* may not have substantial effects on strawberry ripening; however, the fungus was also shown to induce genes involved in the biosynthesis of other plant hormones such as ABA. Moreover, *B. cinerea* can synthesize and secrete ABA that functions as a virulence factor (Siewers *et al.* 2004, 2006). Besides hormones, increased oxidative reactions caused by the pathogen may influence ripening progression (Bianco *et al.*, 2009; Wang *et al.*, 2017a).

## Mechanisms of Defence and Avoidance Against *Botrytis cinerea*

Defence mechanisms can be divided into preformed and induced defences. In strawberries, preformed defence compounds are especially abundant in the unripe stage, as reviewed in the section on quiescence of *B. cinerea*. Even though plants accumulate defence compounds, *B. cinerea *has mechanisms to cope with these metabolites by efflux and detoxification of inhibitory substances. ATP‐binding cassette (ABC) transporters are used by *B. cinerea *to facilitate the efflux of antifungal compounds, such as stilbenes (Schoonbeek *et al.*, 2002; Schoonbeek *et al.*, 2001). *B. cinerea* is capable of detoxifying inhibitory substances, like epicatechin by secretion of laccases (Amil‐Ruiz *et al.*, 2011; Staples and Mayer, 1995). Active *B. cinerea* infections can result in a reduction of specific secondary metabolites. It has been reported that levels of flavan‐3‐ol, benzoic acid and phenylpropanoids drop in *B. cinerea*‐infected strawberries (Nagpala *et al.*, 2016).

Strawberries respond to *B. cinerea *infection by triggering defences. In some cases, preformed and induced defences can overlap such as in the case of PGIPs. An endogenous PGIP appears to be constitutively expressed in fruit from various strawberry cultivars (Mehli *et al.*, 2004). However, this PGIP and six additional ones show higher expression levels upon infection with *B. cinerea* (Schaart *et al.*, 2005). Overexpression of *FaPGIP1a* and *FaPGIP2a* in cisgenic plants conferred enhanced resistance to grey mould (Schaart, 2004). Other enzymes induced by *B. cinerea* infections are chitinases. Expression of the chitinases *FaChi2‐1* and *FaChi2‐2* peaked 16 hpi in *B. cinerea‐*infected strawberries (Mehli *et al.*, 2005). Furthermore, heterologous expression of *Phaseolus vulgaris* chitinase cH5B in strawberry resulted in higher resistance to infection (Vellicce *et al.* 2006). Another study demonstrated that application of heat‐inactivated cells of the yeast *Aureobasidium pullulans* promoted tolerance to *B. cinerea* in strawberries (Adikaram *et al.*, 2002). This primed resistance is probably due to the fruit's perception of chitin from the yeast leading to induction of chitinases or other plant immune responses. Moreover, fruit defence responses may be primed using mechanical stimulation as it was reported for strawberry leaves (Tomas‐Grau *et al.*, 2017).

Induced defences include accumulation of secondary metabolites and ROS. For instance, strawberries accumulate proanthocyanins around infection zones possibly to restrict fungal growth (Feucht *et al.*, 1992; Jersch *et al.*, 1989). The surroundings of infection sites generally display higher ROS production (Tomas‐Grau *et al.*, 2017). ROS can serve as an effective defence against pathogens but also can lead to cell death, which is considered beneficial for necrotrophic fungi (Prusky and Lichter, 2007). *B. cinerea *itself produces ROS to induce host cell death, deplete plant antioxidants and increase lipid peroxidation (van Kan, 2006). It is therefore interesting that, in unripe tomato fruit ROS production leads to resistance against *B. cinerea*, whereas in ripe fruit it seems to promote susceptibility (Cantu *et al.*, 2008, 2009). Future research will likely shed more light on the role of ROS in induced defences of strawberry fruit.

Basal immunity is activated upon fungal infection. Degradation of fruit cell wall pectins can produce demethylated oligogalacturonides that trigger basal immune responses (Amil‐Ruiz *et al.*, 2011). Expression of the *F.  x ananassa* pectin methylesterase 1 *FaPE1* in *Fragaria vesca* resulted in reduced methyl‐esterification of oligogalacturonides in fruit. This reduced esterification activated basal defences via the salicylic acid (SA) signalling pathway that led to a higher resistance to *B. cinerea *(Osorio *et al.*, 2011). Involvement of SA signalling in responses against *B. cinerea *was previously suggested when strawberry plants and fruit treated with SA showed decreased postharvest decay (Babalar *et al.*, 2007). *B. cinerea* can suppress the expression of plant defence responses by hijacking the host sRNA regulatory pathways (Weiberg *et al.*, 2013). In strawberry fruits, *B. cinerea* infections can alter the expression of microRNAs involved in the regulation of defence genes, including the plant intracellular Ras group‐related LRR protein 9‐like gene (Liang *et al.* 2018). Interestingly, *B. cinerea* can also take up plant sRNAs during its interaction with the host. For instance, transgenic plants expressing sRNA that targets *B. cinerea* DCL1 and DCL2 show significantly reduced fungal growth in strawberries (Wang *et al.*, 2016). The suppression of fungal growth via host sRNA is not well understood, and it is yet to be demonstrated that this mechanism of defence naturally occurs in plants.

## Variation of Quantitative Resistance to *Botrytis cinerea* in Strawberry

The diverse arsenal of infection mechanisms employed by *B. cinerea* explains its extremely wide‐host range. It is therefore not surprising that entirely resistant strawberry genotypes do not exist (Bestfleisch *et al.*, 2015; Bristow *et al.*, 1986). Several authors have analysed field resistance of strawberries to *B. cinerea *by quantifying disease development without artificial inoculation. A multi‐year study of three strawberry cultivars found a significant effect of year, cultivar and cultivar by year interaction on the incidence of *B. cinerea* infections (Rhainds *et al.*, 2002). Moreover, there was a positive correlation between row density and disease. Other studies investigated field resistance in annual winter production systems and found that variation of *B. cinerea *incidence between years was larger than genotype differences within years (Chandler *et al.*, 2004; Seijo *et al.*, 2008). Even though field resistance assessments investigate conditions similar to commercial production, considerable variability between environmental conditions and years can interfere with the detection of genotype differences.

Due to the confounding effects of different non‐genetic variables in field studies, assessment of postharvest resistance to *B. cinerea *infections has been pursued to determine genotype differences between strawberry cultivars or species. A large study of grey mould development during postharvest storage of non‐inoculated fruit reported variation in disease incidence and speed of progression amongst cultivars, but no complete resistance was observed (Lewers *et al.*, 2012). Another approach to reducing environmental effects in disease tests is to inoculate fruit with *B. cinerea *conidia suspensions. Bestfleisch *et al.* (2015) tested quantitative resistance in 107 accessions of wild and cultivated strawberry. In this study, two wild ecotypes of *F. virginiana *showed high resistance to *B. cinerea *infections and slow disease progression. Such high tolerance in wild species was also reported in *B. cinerea*‐inoculated leaves and fruit of *F. chiloensis* accessions from Chile (González *et al.*, 2009). In these wild accessions, *B. cinerea *grew much slower. Comparative studies of disease progression indicated that fruit from the cultivar Chandler developed lesions at 24 hpi, while fruit from an *F. chiloensis* ecotype developed symptoms at 72 hpi (González *et al.*, 2013). Fruit were entirely covered with mould at 6 days post‐infection (dpi) for the cultivar Chandler and at 9 dpi for the *F. chiloensis* ecotype.

Considering that some accessions, particularly wild ecotypes, show reduced grey mould incidence and progression, there might be genetic sources of resistance against *B. cinerea* that could be used to increase resistance in strawberry. However, information about resistance mechanisms is mostly based on assumptions or empirical data. Differences in ripening patterns have been suggested as a potential explanation for resistance. For instance, some strawberries ripen from inside to outside, leaving the skin, which is the entry point of infections, unripe and thus resistant for a longer time (Jersch *et al.*, 1989). Some more tolerant cultivars remain white or unripe around the calyx (white shoulders), which is where many *B. cinerea *infections tend to initiate. Another mechanism of resistance could be the presence of fungal inhibitors or the induction of PR proteins. *FcPR5* and *FcPR10* are highly induced in resistant *F. chiloensis* accessions when compared to commercial *F.  x ananassa* cultivars (González *et al.*, 2013). Based on sequence homology, *FcPR5 *probably possesses antifungal activity, and *FcPR10 *is likely a ribonuclease. These findings reflect that even though efforts have been made to explore resistance mechanisms of strawberry to *B. cinerea,* very little is known. Therefore, more research is necessary to better understand the biology of strawberry interactions with *B. cinerea* infections using diverse germplasm accessions.

## Current and New Management Approaches for *Botrytis cinerea* in Strawberry Production

Many disease management strategies have been implemented for the control of *B. cinerea* in strawberry as further described below. However, even combined approaches are only capable of reducing disease incidence and severity but cannot completely prevent or eliminate grey mould in strawberries (Feliziani and Romanazzi, 2016).

### Agronomic and horticultural practices

Historically, *B. cinerea* infections in strawberry production have been managed by agronomic and horticultural practices, such as removal of senescent plant material to avoid inoculum buildup (Daugaard, 1999). Preventing contact of fruit with soil (e.g. covering the planting beds with polyethylene foils) is another common practice to avoid *B. cinerea* infections, as most of the inoculum is present on the ground and soil moisture promotes conidia germination (Daugaard, 1999). Selecting the right irrigation system could help reduce grey mould incidence; mainly, the use of drip irrigation and micro‐sprinklers results in limited inoculum spread and reduction of water films on the fruit (Dara *et al.*, 2016; Terry *et al.*, 2007). As canopy characteristics influence microclimates (e.g. humidity, airflow, contact between plants), nitrogen fertilization can lead to dense canopies and favour grey mould (Daugaard, 1999). Similarly, shorter plant spacings promote higher incidence of *B. cinerea *in the field (Legard *et al.*, 2005). Additionally, plastic tunnels can avoid airborne inoculum and *B. cinerea* incidence is lower in non‐fungicide treated tunnels than in fungicide treated fields (Xiao *et al.*, 2001), but tunnels favour powdery mildew and complicate harvest. In summary, cultural practices are essential to limit preharvest *B. cinerea* infections of strawberries, especially in organic agriculture.

### Fungicides

In modern production, pesticide applications are the most common management practice for *B. cinerea *control (see Table 1). In the previous two decades, the main pesticides used in strawberry production against *B. cinerea* belonged to the Fungicide Resistance Action Committee (FRAC) Groups 1 and 2, as well as captan (Sutton, 1990; Wedge *et al.*, 2007, 2013). However, due to increasing fungicide resistance and new legal restrictions, producers have been forced to diversify their fungicide regimen (Vellicce *et al.*, 2006; Wedge *et al.*, 2013). The frequency and timing of fungicide applications are crucial for *B. cinerea* control. One application of fenhexamid (FRAC 17) at anthesis can be as efficient as multiple weekly applications (Mertely *et al.*, 2002). Additionally, alternation and combination of different fungicides with different modes of action are recommended (Wedge *et al.*, 2007).

**Table 1:** Registered fungicides for control of *Botrytis cinerea* in strawberry production.

FRAC code	FRAC group	Target site	Target action	Risk of resistance	Example
FRAC 1	Benzimidazoles	β‐tubulin assembly in mitosis	Cytoskeleton	High	Benomyl
FRAC 2	Dicarboximides	MAP/histidine kinase in osmotic signal transduction (os‐1, Daf1)	Signal transduction	Medium to high	Iprodione
FRAC 7	Succinate dehydro‐genase inhibitors	Succinate dehydrogenase	Respiration	Medium to high	Boscalid
FRAC 9	Anilinopyrimidines	Methionine synthesis	Amino acid and protein synthesis	Medium	Cyprodinil
FRAC 11	Quinone outside inhibitors	Cytochrome BC1 at Qo Site	Respiration	High	Azoxystrobin
FRAC 12	Phenylpyrroles	MAP/histidine kinase in osmotic signal transduction (os‐2, HOG1)	Signal transduction	Low to medium	Fludioxonil
FRAC 17	Sterol biosynthesis inhibitors class III	3‐Keto reductase in C4 de‐methylation	Inhibition of sterol biosynthesis in membrane	Low to medium	Fenhexamid
FRAC M03	Dithiocarbamates and relatives	Multi‐site mode of action		Low	Thiram
FRAC M04	Phthalimides	Multi‐Site Mode of Action		Low	Captan

Resistance of *B. cinerea *to fungicides is a real challenge in horticulture and fungicide resistance profiles can shift considerably even within a single season (Cosseboom *et al.*, 2019; Konstantinou *et al.*, 2015; Leroch *et al.*, 2013; Wedge *et al.*, 2007). A screen of 13 *B. cinerea *isolates in Louisiana (USA) showed that all were partial to full resistance to FRAC 1 fungicides, and several of the isolates also had different levels of resistance to FRAC 2 fungicides (Wedge *et al.*, 2013). A larger survey of 1890 *B. cinerea *isolates (189 fields in 10 states of the USA) revealed that 7 isolates from different locations were resistant to all single‐action site FRAC fungicides groups that are registered for *B. cinerea* control (Fernández‐Ortuño *et al.*, 2015). *B. cinerea* resistance to fungicides is usually associated with overexpression of efflux transporters or with modification of fungicide targets. These resistance mechanisms are acquired via mutations and recombination that occur frequently in *B. cinerea *due to heterokaryosis, sexual reproduction and the presence of abundant transposable elements in its genome (Konstantinou *et al.*, 2015). Efflux of fungicides or accumulation of altered fungicide targets has also been shown to lead to multi‐resistances (Konstantinou *et al.*, 2015; Rupp *et al.*, 2017). The presence of resistant isolates against the most common multi‐action site fungicides reinforces the need for innovative management practices. A new generation of RNA‐based fungicides has been proposed, which relies on the application of sRNA or dsRNAs that target *B. cinerea* virulence genes to reduce fungal infections in strawberries (Wang *et al.*, 2016). However, these RNA‐based fungicides remain far from commercialization, which is why fungicide resistance management such as mixture and rotation of different fungicides or testing local isolates for resistance is necessary (Hahn, 2014).

### Biological control

To date, *B. cinerea *biocontrol products are mostly *Bacillus subtilis*‐based, but their use in commercial strawberry production is limited because of their insufficient applicability in the field or supply chain (Pertot *et al.*, 2017). Nevertheless, there is social and scientific interest in using biocontrol against *B. cinerea *as an alternative to chemical pesticides. Isolates of *Colletotrichum gloeosporioides, Epicoccum purpurascens, Gliocladum roseum, Penicillium sp., Trichoderma sp.* have displayed high efficiency in controlling *B. cinerea* and were reported to reduce grey mould incidence on strawberry stamens by 79%–93% and on fruit by 48%–76% (Peng and Sutton, 1991). Interestingly, in some experiments, the efficiency of biocontrol by these organisms exceeded the efficacy of control via the fungicide captan. Similar results were obtained for other microbes, such as the yeasts *A. pullulans* (Adikaram *et al.*, 2002) and *Candida intermedia* (Huang *et al.*, 2011), the filamentous ascomycete *Ulocladium atrum* (Boff, 2001; Boff *et al.*, 2002a, 2002b), or the bacterium *Bacillus amyloliquefaciens* (Sylla *et al.*, 2015).

Biocontrol via microbes can work via different modes of action, including competition for nutrients, secretion of antibiotic compounds and induction of host defence mechanisms like the up‐regulation of chitinase and peroxidase activity (Adikaram *et al.*, 2002; Ippolito *et al.*, 2000; Lima *et al.*, 1997; McCormack *et al.*, 1994). Because biocontrol of *B. cinerea *relies on a variety of mechanisms, the most significant effects are observed when different organisms are applied in combination (Sylla *et al.*, 2015; Xu and Jeger, 2013). As alternative to applying living microbes, use of extracts or volatiles derived from biocontrol microbes has been suggested (Huang *et al.*, 2011). Use of non‐synthetic antifungal substances, like phenol‐rich olive oil mill wastewater, has also been reported to control *B. cinerea *growth *in vitro* and on strawberries (Vagelas *et al.*, 2009). However, these approaches are not implemented on a commercial scale due to high costs compared to the conventional *B. cinerea* control

### Postharvest treatments

It is common practice to handpick strawberries and place them into clamshells in the field, in order to reduce wounding and bruising of the fruit. Rapid and constant cooling of strawberries at temperatures below 2.5 ºC is another critical strategy to reduce or inhibit reactivation of *B. cinerea* quiescent infections (Nunes *et al.*, 1995). Often, strawberries are also stored in modified atmospheres, which are generally low in oxygen and high in carbon dioxide to slow down metabolic processes, senescence and fungal decay (Feliziani and Romanazzi, 2016). Relative humidity during storage is usually kept around 85%–90% to prevent dehydration of fruit, but limit fungal growth (Almeida *et al.*, 2015).

Novel postharvest treatments of strawberries have been suggested to prevent *B. cinerea* infections during storage. Examples are edible fruit coatings of chitosan, silk fibroin or methylcellulose that prevent water loss and can include antifungal compounds (Marelli *et al.*, 2016; Nadim *et al.*, 2015; Romanazzi *et al.*, 2017). MeJA treatment to induce fruit defence mechanisms (Zhang *et al.*, 2006), ultraviolet and visual light treatment (Saks *et al.*, 1996), enrichment of storage atmosphere with chlorine or ozone (Avis *et al.*, 2006; Nadas *et al.*, 2003), and soft mechanical stimulation (Tomas‐Grau *et al.*, 2017) have also been tested as alternative treatments. Most of these approaches are still in an experimental stage or not yet adaptable to commercial settings.

## Looking into the Future: Improving Strawberry Resistance to *Botrytis cinerea*

Several aspects of the genetics of resistance to *B. cinerea* are unclear in strawberry. Significant phenotypic variation of incidence or severity of grey mould has been reported; however, *F*.  x *ananassa* genotypes appear to be universally susceptible and complete resistance has not been observed (Bestfleisch *et al.*, 2015). Substantial genotypic variation has not been documented and the heritability of resistance to *B. cinerea *is unknown. Mild phenotypic differences in fruit resistance levels reported in various postharvest studies (Bestfleisch *et al.*, 2015; Lewers *et al.*, 2012) indicate that genetic variation for resistance may be limited and that its heritability is low. A contributing factor is the intrinsic characteristics of the pathogen, its broad host range, diverse ways of infection and necrotrophic lifestyle, which explain the absence of a gene‐for‐gene resistance of strawberry to *B. cinerea* (Amil‐Ruiz *et al.*, 2011). Therefore, breeding for escape and tolerance, which includes physiological and biochemical traits, is a more practical option (Elad and Evensen, 1995). While limited in scale and scope, earlier studies strongly suggest that the incidence and progression of *B. cinerea* infections differ between cultivars with soft fruit and those with firm fruit (Barritt, 1980; Gooding, 1976). Hence, previously reported differences amongst cultivars could be the result of the pleiotropic effects of selection for increased fruit firmness and shelf life and the associated developmental and ripening changes, as opposed to direct genetic gains in innate resistance to the pathogen.

As discussed, fruit firmness is an important trait associated with resistance to *B. cinerea* (Hancock *et al.*, 2008; Terry *et al.*, 2004). The strawberry germplasm displays natural variation for fruit firmness and developing cultivars with firmer fruit is an important aim in breeding programmes (Hummer *et al.* 2011; Salentijn *et al.*, 2003). Changes in flower morphology could also enhance tolerance to *B. cinerea*. In strawberry, most *B. cinerea *infections in fruit appear to originate from primary infections of flowers or secondary infections caused by direct contact with infected flower parts (Bristow *et al.*, 1986; Jarvis, 1962; Powelson, 1960). It was reported that removal of stamen and petals result in lower grey mould incidence (Jersch *et al.*, 1989; Powelson, 1960). Faster abscission of flower parts, especially petals, has the potential to aid the escape of strawberries from *B. cinerea *infections (Elad and Evensen, 1995). Similarly, plants with pistillate flowers (i.e. flowers with pistils but no stamen) have a lower grey mould incidence in fruit (Bristow *et al.*, 1986; Elad and Evensen, 1995). *B. cinerea *growth inhibition in stamens is reported to vary within the strawberry germplasm, potentially due to differences in their biochemical composition (Bristow *et al.*, 1986). Similarly, antifungal compounds in fruit can prevent or limit *B. cinerea *infections. Several reports indicate that anthocyanin accumulation contributes to tolerance of strawberries to *B. cinerea* (Jersch *et al.*, 1989; Saks *et al.*, 1996). Anthocyanins do not just improve tolerance to grey mould but also provide health benefits (Terry *et al.*, 2004). Breeding for higher anthocyanin content in strawberries is possible and facilitated by existing variation in the germplasm (Fredericks *et al.*, 2013; Jing, 2012). Inducing anthocyanin accumulation in flowers could also help to limit flower infections.

As breeding for higher *B. cinerea* tolerance will be tedious and likely will not result in complete resistance, complementary approaches should be considered. Currently, no genetically modified strawberry cultivars are commercially grown; however, several reports show great potential to improve tolerance to grey mould via trans‐ or cis‐genesis. For example, the expression of chitinases or PGIPs from other organisms in strawberries can prevent or slow down fungal infections (Powell *et al.*, 2000). Another potential transgenic approach is to increase fruit firmness by altering the expression or activity of pectin degrading enzymes, such as PL or PG (Jimenez‐Bermudez *et al.*, 2002). The existing natural variation of PL expression levels and activity in the cultivated strawberry germplasm could be used for cisgenic approaches. Increasing phenolic levels in strawberries by genetic modifications can also be explored as the transcription factor MYB10 was identified as a regulator of anthocyanin levels in strawberries (Lin‐Wang *et al.*, 2010; Lin‐Wang *et al.* 2014; Medina‐Puche *et al.*, 2014). Transgenic plants (both *F.  x ananassa* and *F. vesca*) with ectopic overexpression of MYB10 show elevated anthocyanin levels throughout the entire plant (Lin‐Wang *et al.*, 2010); however, the resistance of these plants against *B. cinerea* have not been tested. In summary, these novel breeding approaches should be supported by integrative management strategies including horticultural and agronomic practices, and potentially biocontrol, to achieve maximum control of the disease.

## Conclusion

Many details about grey mould of strawberries are still poorly understood. Future research is necessary to characterize the genetic pathways and biochemical components that are involved in strawberry‐*B. cinerea* interactions. Molecular analyses of the infection process and the physiological causes for the failure of host defences should provide a basis to develop robust solutions against the disease, or at least provide information for control strategies that are likely to fail and therefore be discouraged. Furthermore, current disease management needs to be re‐evaluated to cope with increasing restrictions and lack of efficacy of fungicides. Investigations on biocontrol approaches and pre‐ and postharvest treatments are necessary to manage grey mould. On the other hand, breeding for escape and tolerance against *B. cinerea* can be a feasible approach for commercial varieties. Research on genetic modifications of strawberry that restrict *B. cinerea* infections could also be used for guiding conventional breeding efforts or developing new varieties once the market is ready for their acceptance.
